# Methodological Considerations in Evaluating Mental Health Apps: Ensuring Reliability and Patient Safety

**DOI:** 10.2196/85329

**Published:** 2025-11-19

**Authors:** Harikrishnan Balakrishna

**Affiliations:** 1 Department of Health Services, Family Health Centre, Kilikolloor, Government of Kerala, Mangadu PO, Kollam, 691015, India, 91 8078379594

**Keywords:** mental health apps, mHealth, review of apps, smartphone apps, MHApps for Indian users, India, mobile phones

We read with great interest and appreciate Mehrotra et al [1] for their systematic review of mental health apps accessible to Indian users. Their assessment of 350 apps provides valuable insight into the current digital mental health landscape in India. Still, several methodological and reporting considerations require clarification. This letter addresses 3 key areas: interrater reliability, sampling scope, and patient safety considerations.

To establish interrater reliability, the Mobile Application Rating Scale (MARS) was developed and validated with independent dual ratings and calculation of intraclass correlation coefficients (ICCs) [2]. Mehrotra et al [1] describe efforts like joint training and expert validation of a small subsample but divide the remaining apps among 4 reviewers for independent evaluation. ICC values for the main ratings were not reported, an approach that deviates from the established MARS methodology [2]. A past study demonstrated that when MARS is applied without strict dual-rater methodology, interrater agreement can be low (Krippendorff α=0.29), indicating high variability among reviewers [3]. Without ICC data, it is difficult to determine whether differences in app quality scores reflect their true variation or rater inconsistency. Reporting ICCs for a random subsample or acknowledging this limitation would improve interpretability for clinical application.

Regarding sampling scope, World Health Organization (WHO) guidance emphasizes transparent reporting and clear description of inclusion criteria for digital health interventions to support accurate interpretation and generalizability [4]. The study assessed only free apps and freely accessible portions of paid apps, yet its conclusions are framed as applying to all “mental health apps available to Indian users.” Premium apps often differ from free ones in professional input, empirical validation, and privacy safeguards. The finding that 65% of apps lacked professional input and only 11% cited empirical research [1] may therefore not represent the entire app ecosystem. This approach limits its generalizability and could mislead policymakers and clinicians relying on evidence-based recommendations. Hence, conclusions should be categorically limited to the free or freemium app marketplace or, in future work, expanded to include paid apps.

Regarding patient safety considerations, WHO guidance emphasizes that systematic evaluations of digital health interventions should transparently identify and report potential harms to support safe implementation and evidence-based decision-making [4]. The authors identified apps with potentially harmful features, such as misleading claims or alarming feedback without crisis support, but did not indicate whether these concerns were addressed or reported in any way. Vulnerable patients experiencing mental health crises could have worsened outcomes when exposed to such inadequately designed assessment tools [5]. Empirical evidence shows that adverse events in consumer-facing mental health apps are underreported and may pose significant risks to users [5]. Highlighting such risks ensures that clinicians, policymakers, and end users are aware of potential harms when translating review findings into practice, without implying that reviewers are responsible for regulatory action.

Clarifying interrater reliability, explicitly defining app sampling scope, and transparently reporting safety-relevant issues strengthen the interpretability and applicability of systematic reviews in digital mental health. Methodological limitations, such as inconsistent rating procedures or selective sampling, reduce confidence in app quality scores and limit generalizability. While patient safety considerations are primarily reporting issues, systematically highlighting potentially harmful apps enhances the practical utility of reviews and supports safe evidence-based decisions.

## Abbreviations

ICC: intraclass correlation coefficient
MARS: Mobile Application Rating Scale
WHO: World Health Organization
